# Physical Activity May Be Associated with Conditioned Pain Modulation in Women but Not Men among Healthy Individuals

**DOI:** 10.1155/2017/9059140

**Published:** 2017-09-26

**Authors:** Yukiko Shiro, Tatsunori Ikemoto, Yuta Terasawa, Young-Chang P. Arai, Kazuhiro Hayashi, Takahiro Ushida, Takako Matsubara

**Affiliations:** ^1^Department of Physical Therapy, Faculty of Rehabilitation Sciences, Nagoya Gakuin University, Nagoya, Japan; ^2^Multidisciplinary Pain Center, Aichi Medical University, Aichi, Japan; ^3^Institute of Physical Fitness, Sports Medicine and Rehabilitation, School of Medicine, Aichi Medical University, Aichi, Japan; ^4^National Hospital Organization, Higashi Nagoya National Hospital, Aichi, Japan; ^5^Department of Rehabilitation, Faculty of Health Sciences, Nihon Fukushi University, Aichi, Japan

## Abstract

**Background:**

Conditioned pain modulation (CPM), a phenomenon also known as diffuse noxious inhibitory control, is thought to be affected by various factors, including sex and level of physical activity. However, the involvement of these factors in CPM remains unclear.

**Methods:**

Eighty-six healthy young subjects (M/F, 43/43) participated in this study. Participants were assessed on the basis of their mechanical pressure pain threshold (PPT), CPM response, body mass index (BMI), basal metabolic rate (BMR), and duration of moderate-to-vigorous physical activity (MVPA) over a week, using a motion counter. Response to CPM was evaluated as PPT during painful cold stimulation relative to baseline PPT.

**Results:**

Men showed significantly higher baseline PPT than women; however, this difference was no longer significant after controlling for confounders. Stepwise multiple linear regression analyses revealed BMR to be a significant contributor towards baseline PPT in the entire study population. In contrast, although there were no significant contributors to CPM response among men and in the overall study group, MVPA was positively associated with CPM response among women (*β* = 0.397).

**Conclusions:**

These results suggest that, among healthy young individuals, CPM response may be associated with moderate-to-vigorous physical activity in women but not in men.

## 1. Introduction

Pain is a subjective experience, and several excitatory and inhibitory endogenous mechanisms are known to influence the transmission of noxious stimuli. Recent studies have examined endogenous pain modulatory processing within the central nervous system by using an experimental test termed conditioned pain modulation (CPM). CPM, a phenomenon also known as diffuse noxious inhibitory control (DNIC), refers to the process whereby a noxious stimulus applied to one body part results in decreased pain perception from another noxious stimulus applied at a distal body part [1, 2].

Previous studies have reported that CPM is affected by various factors, particularly sex [3, 4] and level of physical activity [5]. Furthermore, chronic pain conditions appear to cause a decrease in CPM [6, 7], and these findings support the major characteristics of chronic pain. For example, the prevalence of chronic pain is higher among women than among men [8], and common chronic pain conditions including fibromyalgia, migraine, temporomandibular disorders, and rheumatic diseases are more prevalent among women than among men [9]. Research addressing the effect of sex on one-dimensional pain sensitivity measures has mostly produced consistent results indicating that women demonstrate a higher pain sensitivity than men [10, 11]. In addition, some recent studies have reported a less efficient pain inhibitory capacity in women compared to that in men [12]. In contrast, other studies have reported no difference in the magnitude of CPM responses according to sex [13, 14]. Popescu et al. found that sex-wise differences in DNIC depend both on experimental methodology and on the modes used for measuring the effect [3].

It is also well known that inactivity is a risk factor for development of chronic pain [15]. Moreover, increase in physical activity attenuates the severity of symptoms in patients with chronic pain [16, 17]. Accordingly, guidelines for treatment of musculoskeletal pain include recommendations for exercise for preventing progression to chronic pain [18]. Pain relief, which is the overall beneficial effect of physical activity, can be achieved through activation of endogenous pain inhibitory mechanisms [19]. Furthermore, some studies suggest that engaging in vigorous physical activity might help reduce sensitivity to experimental pain stimulation in healthy adults [20–22]. Conversely, decrease in physical activity and dysfunction of endogenous pain modulation have been reported in patients with chronic pain [23, 24]. Meanwhile, several studies have reported that a relationship exists between level of physical activity and chronic pain among women [25, 26]. These studies have also shown that physical activity is positively related to brain responses implicated in pain modulation, including responses in the dorsolateral prefrontal cortex, dorsal posterior cingulate, and periaqueductal grey in women [26, 27]. Therefore, it might be more important for women than for men to maintain physical activity in order to prevent chronic pain. However, not many studies have investigated the association between pain sensitivity and physical activity of participants using objective devices such as motion counters.

Despite these findings, the dominant factor influencing CPM is still unknown. Previous studies have suggested that men are more active than women during leisure time [28]. The effects of sex and physical activity on CPM have not yet been assessed despite the fact that men have larger body components and greater basal metabolic consumption than women [29, 30]. Lowe et al. reported that female patients with fibromyalgia have lower metabolic rate than healthy control subjects [31], indicating that metabolic rate could be involved in pain modulation. Several studies have indicated high body mass index (BMI) to be associated with chronic pain [32, 33] and pain sensitivity [34]. Thus, basal metabolic rate (BMR) and BMI might be potential confounders in the association of pain sensitivity with sex. Moreover, recent studies have suggested that CPM response could be influenced by ethnic background [35, 36] as well as aging [37, 38].

Additionally, many previous studies have used small sample sizes for statistical analysis for evaluating CPM response, which has also been pointed out by Riley 3rd et al. [39]. Kennedy et al. recently suggested that lack of control for confounding factors and lack of standardization in statistical analysis are common problems affecting the reliability of CPM measurements [40]. Therefore, rigorous eligibility criteria with a sufficient sample size of young healthy individuals would be helpful for elucidating the physiological differences in CPM response between men and women and for clarifying the relationship between CPM response and physical activity.

Altogether, in present study, we hypothesized that (1) although pressure pain threshold (PPT) would be higher among men than among women [10], this difference would be affected more by other possible confounders than by sex; (2) there would be sex-wise differences in CPM response; (3) although physical activity and CPM response would be related, this relationship would more likely be seen in women than in men. To address these hypotheses, we investigated the relationship of pain threshold and CPM response towards pressure pain with sex, BMR, BMI, and physical activity level measured using a motion counter among healthy Japanese individuals.

## 2. Methods

### 2.1. Ethical Approval and Subjects

After receiving approval from the Nagoya Gakuin University Board of Ethics and obtaining written informed consent, we recruited healthy subjects to participate in the present study by means of flyers on a notice board. The inclusion criteria for participation were (1) age between 20 and 29 years and (2) no ongoing pain problems. On the other hand, the exclusion criteria were history of chronic pain conditions and serious health conditions such as neurological diseases (e.g., stroke or hereditary diseases), diabetes, or use of sedatives, analgesics, or other medications.

Minimum sample size was estimated in accordance with three previous hypotheses. According to a previous report, at least 41 subjects per sex are needed for comparison of differences in PPT with sufficient analytical power (>.80) [40]. In terms of the effect of sex on CPM, Bulls et al. [4] performed a CPM study and reported that, on the basis of an effect size of .64, the sample size for a power of >.80 and a two-tailed *α* at a significance level of <.05 for performing a *t*-test required a minimum of 80 subjects (40 subjects per sex). Regarding physical activity, Naugle and Riley 3rd have reported that total physical activity scores (*β* = .431) were predictive of CPM [5] and that 37 subjects per sex are required to detect this correlation coefficient by bivariate regression analysis with a two-tailed *α* of <.05 and a power of >.80. For a priori power analysis, we used the G*∗*power 3 software [41] to determine the sample size for this study.

Finally, we enrolled 86 volunteers (43 men and 43 women; age, 20.9 ± 0.8 years) for participation in this study, which was conducted in three separate intervals, because it was difficult to gather the necessary number of subjects in a single round. Each subgroup participated in the study in March, May, and November 2016.

### 2.2. Participant Characteristics and Physical Activity Measurement

The height and body weight of all participants were measured using scales, and BMI was calculated from these values using the following formula: BMI = weight (kg)/height (m^2^); BMR was calculated using the Harris-Benedict equation [42] as follows:

  Men: BMR = 66.5 + (13.7 × weight [kg]) + (5.00 × height [cm]) – (6.8 × age [years]).
  Women: BMR = 655.1 + (9.56 × weight [kg]) + (1.85 × height [cm]) – (4.68 × age [years]).

Before pain measurement, in order to measure the degree of physical activity for 7 consecutive days, all participants were instructed to wear an accelerometer (Kenz Lifecorder, Suzuken Co., Ltd., Nagoya, Japan) at the waist level throughout the day except during water-related activities (e.g., bathing and swimming) [5]. Kenz Lifecorder is a validated device with good reliability [43]. This motion counter measures acceleration in the vertical (*z*) direction. According to technical details provided by the manufacturer, the device samples acceleration at 32 Hz and assesses values ranging from 0.06 to 1.94 g (1.00 g is equal to the acceleration of free fall). The acceleration signal is filtered by an analogue bandpass filter and digitized. Maximum pulse over 4 s is considered as the acceleration value, and activities are categorized into eleven activity levels, 0.0, 0.5, and 1.0–9.0 (where level 0.0 corresponds to 0.06 g), on the basis of the accelerometric signal pattern.

Consecutive activities performed by the participants were recorded automatically, and physical activity was automatically expressed as sedentary (<level 1.0), light (levels 1.0–3), moderate (levels 4–6), or vigorous (≥level 7). These activity levels were also automatically transformed to the corresponding estimated metabolic equivalents (METs) as follows: level 1.0, 1.8 MET; level 2.0, 2.3 MET; level 3.0, 2.9 MET; level 4.0, 3.6 MET; level 5.0, 4.3 MET; level 6.0, 5.2 MET; level 7.0, 6.1 MET; level 8.0, 7.1 MET; and level 9.0, >8.3 MET. The principle of transformation in this device has previously been described in detail [44].

In the present study, the time spent engaging in moderate-to-vigorous physical activity (MVPA) was considered as the physical activity score for each individual. This was because light activity was regarded as corresponding to less activity than walking at normal speed for young healthy participants [44]. The device computed an exercise value (METs × h/week), which was calculated by the sum of activity levels (≥level 4.0) for every 4 s over 7 days. Finally, the exercise value was defined as the MVPA score for each participant.

Days when the device was not worn for 4 consecutive hours on the same day were excluded from analysis. It was easy to distinguish between sleep motion (variable within level 0.0 to level 0.5) and wearing no device (completely level 0.0) on the recorded data.

### 2.3. Pain Measurement

The pain measurement session consisted of a training and test session on the same day. In the training session, participants received information about and experienced the entire experimental procedure. The test session was performed immediately after all participants had finished the training session.

Subjects sat in a fixed chair and placed their dominant forearm on the desk. On the dominant forearm, PPT was evaluated at a site over the extensor carpi radialis brevis muscle, 2 cm distal to the lateral epicondyle. The mechanical force transmitted to the muscle was measured using a calibrated mechanical pressure algometer (Digital Force Gage, AIKOH, Osaka, Japan). The rubber tip of the algometer was 1 cm in diameter. The algometer was applied to the testing site, and pressure was gradually increased by approximately 5 N/s during the test. Participants were instructed to respond immediately when they felt pain because of the pressure applied, at which point pressure testing was stopped and the results were automatically recorded. All mechanical stimuli in this study were applied by a single examiner (Y. S.) who had practiced extensively using an electric balance to ensure that the algometer was successfully applied to the participants in a constant manner.

For evaluating the level of CPM response, algometric assessment on the dominant dorsal forearm provided phasic stimulus, while the cold pressor task was used for application of tonic conditioning stimulus [45]. Baseline PPT was measured prior to immersion of the nondominant hand in a cold-water bath (10°C [12] for 1 min) of up to whole-hand depth. After 30 s of cold-water immersion of the nondominant hand and immediately after immersion for 1 min, the algometer was again applied on the dominant dorsal forearm for quantifying CPM response relative to the baseline level. These time points were used because a previous study had indicated that the duration of the conditioning stimulus was commonly between 30 s and 2 min for a cold-water bath [46]. In this study, PPT during cold-water immersion of the nondominant hand was adopted as the conditioned PPT, because CPM response has been shown to be significantly greater during application of conditioning stimulus than immediately after [47, 48].

Baseline PPT values were used for comparison of PPT between men and women. Analysis of CPM response was accomplished using methods described by previous studies, which had suggested quantification of CPM response as percent change from baseline to conditioned PPTs [1]. Accordingly, the change ratio for PPT within each trial was calculated by the following formula:

  CPM response = (conditioned PPT – baseline PPT)/baseline PPT.

### 2.4. Data Analysis

All data were analyzed using SPSS version 20 (IBM Corp., Armonk, NY). Continuous data were presented as mean and standard deviation. The Shapiro-Wilk test was used to investigate whether data were normally distributed. Comparisons between the two groups were performed using Student's *t*-test or Mann–Whitney *U* test, depending on the data distribution.

Subsequently, variables (such as sex, BMI, BMR, and MVPA) that exhibited a potential correlation with PPT or CPM response at an *α* level of 0.1 in univariate analysis were selected as candidates for multivariate analysis. Multiple regression analysis (forward stepwise selection) was performed to predict and compare the overall effect of the confounders on PPT and CPM response according to sex. A *p* value of <.05 was considered statistically significant.

## 3. Results

Table 1 shows the difference in each variable between the sexes. As expected, BMR was higher among men than among women. There was no significant difference in MVPA between men and women. In pain-related measures, the change ratio for PPT was larger during cold-water immersion (i.e., CPM response) than immediately after CPM among both sexes (men: *p* < 0.001; women: *p* = 0.005). Men showed higher baseline PPT than women, but there were no significant differences according to sex in CPM response or change ratio for PPT immediately after CPM.

**Figure 1:**
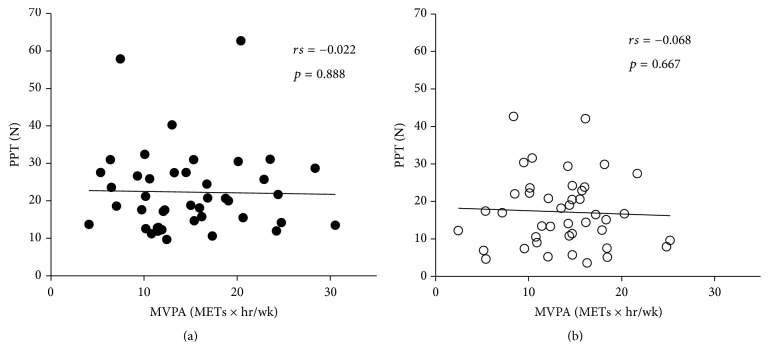
Correlation between PPT and MVPA among (a) men and (b) women. PPT: pressure pain threshold; MVPA: moderate-to-vigorous physical activity.

**Figure 2:**
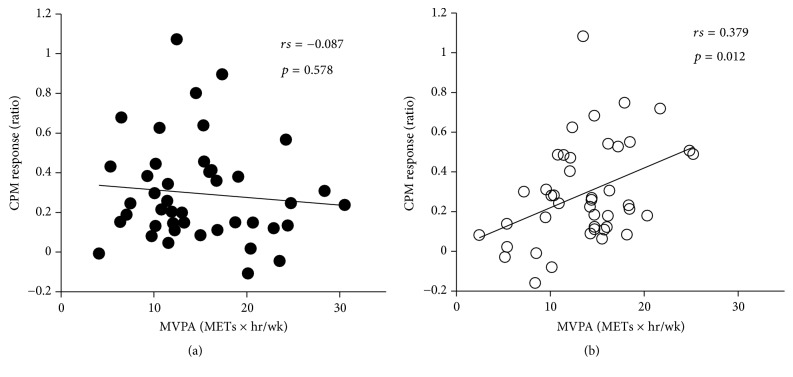
Correlation between CPM response and MVPA among (a) men and (b) women. CPM: conditioned pain modulation; MVPA: moderate-to-vigorous physical activity.

The results of stepwise multiple linear regression analysis revealed that BMR was a significant predictor of PPT in the overall study population. However, PPT was not correlated with BMI, BMR, or MVPA in either sex (Table 2 and Figure 1). On the other hand, although there were no predictors of CPM response in the overall study population or among men, MVPA was moderately correlated with CPM response among women (Table 3 and Figure 2).

## 4. Discussion

This study investigated the relationship of pain threshold and CPM response towards pressure pain with sex, metabolic rate, and moderate-to-vigorous physical activity among healthy young Japanese individuals, using quantitative assessment tools. The major findings of this study were as follows: (1) an interrelationship between PPT and sex was observed, as expected; however, this relationship was no longer significant after controlling for confounders; BMR might be potentially associated with PPT; (2) contrary to our expectations, CPM response did not vary according to sex; and (3) MVPA showed a positive moderate correlation with CPM response among women but not in the overall study population or among men.

Interestingly, although the present results revealed that men might have a higher PPT than women, which is consistent with the current body of evidence [10], this difference was no longer significant after controlling for possible confounders such as BMI, BMR, and MVPA. Our results also indicated that PPT was influenced more by BMR than by sex in each individual. To date, research relevant to the relationship between pain sensitivity and BMR has been scarce, despite the fact that hypothyroidism, a condition of decreased metabolism, is known to be associated with fibromyalgia [49] and pain sensitivity [50]. Our results imply that BMR should be considered as a potential confounder when investigating human pain sensitivity.

Despite the use of many different methodological designs, CPM response is regarded as a complex phenomenon, affected by various predictors [51]. According to the review by Hermans et al. [51], many studies have addressed the influence of sex on CPM; however, 9 of 15 studies found no difference in CPM response according to sex, while the rest reported that men had better CPM function than women. The authors concluded that it remained unclear whether or not sex has any influence on CPM. The present results, too, suggest that there was no difference in CPM effect according to sex. In other words, the functioning of the endogenous pain inhibitory system is not influenced exclusively by sex among healthy Japanese subjects. Although these results are not consistent with the latest data presented by Skovbjerg et al., who demonstrated in a large sample population that women show less efficient CPM than men [52], this inconsistency may be attributed to subject characteristics. That is, it would be difficult to detect sex-wise differences pertaining to CPM if women in the sample population tended to be active (as in the present study) and not inactive (as previously reported) [28].

In the present study, we found that, in women, low MVPA might cause a decrease in CPM response even among healthy young individuals; however, no such relationship was observed among men or in the overall study population. Although recent studies have indicated that greater physical activity is associated with more efficient CPM in healthy adults [5, 53, 54], one study implied that the difference in CPM effect between active and less-active subjects seemed to be greater among women than among men [53]. Previous neuroimaging studies have reported that, in female patients with fibromyalgia, physical activity is positively related to brain responses implicated in pain modulation, including those associated with the dorsolateral prefrontal cortex, dorsal posterior cingulate, and periaqueductal grey [26, 27], which partly supports the results of our study.

The effect of possible confounders and methods of statistical analysis on the present results must also be considered. Although a considerable number of studies have investigated sex-wise differences in many pain modalities, there appears to be a lack of potential confounders. Indeed, age, sex, genetic factors, ethnicity, catastrophizing, anticipation, and physical activity level have been discussed as potential confounders of the CPM effect [40, 51]. For example, Weissman-Fogel et al. identified sex-wise differences in the CPM effect by bivariate comparison; however, after correction for catastrophizing, these differences were no longer significant [55]. The present study included sex, BMI, BMR, and MVPA as potential confounding factors for PPT and CPM response. The results of multiple regression analysis revealed a possible relationship between MVPA and CPM response among women (*β* = 0.397), which is consistent with the findings of Naugle and Riley 3rd [5]. However, the adjusted *R*^2^ in this model was 0.137, which suggested that only approximately 14% of the CPM effect was explained by physical activity within this model. Furthermore, the *p*-trend depends both on correlation coefficients and on sample size; it should never be used as a measure of strength of an association [56]. Therefore, sample size has a strong influence on the quality of correlations. For instance, when a significant correlation between parameters is obtained with a small number of subjects, supplementing this sample with another population that does not exhibit a significant correlation could result in nonsignificance upon analysis of the entire population.

Additionally, several limitations have to be considered in the present study. First, physical activity was measured using a single type of motion counter, the Lifecorder, which calculated the activities of the participants. Since the measurements collected in this study relied only on this uniaxial accelerometer, we cannot be certain that our results would be consistent with those obtained using other types of sensors. Previous studies have evaluated physical activity using triaxial accelerometers [53, 54]; however, some authors have reported significant differences in activity results between uniaxial (i.e., Lifecorder) and triaxial accelerometers [57]. Second, since we only measured MVPA, light activity and sedentary time were not included in our analysis. Additionally, the study period for measuring the activity of participants was limited to only 1 week. Therefore, the long-term effects of physical activity on CPM response are not clear. Third, PPT was only evaluated at one point on the dominant arm, and the CPM effect was evaluated only by means of the cold pressor stimulus. Wider areas of the body should be evaluated using different conditioning stimuli in future studies. Fourth, although previous studies have suggested that CPM response might decrease with age [37, 38], this study only involved young men and women. Further research is needed to test the influence of predictive variables on CPM response among older adults selected using rigorous eligibility criteria. Fifth, participants in this study were healthy volunteers. Thus, the generalizability of the present results among subjects with chronic pain conditions is limited. Last, we did not consider the influence of psychological factors in this study, although a recent study has demonstrated that pain catastrophizing might be related to disruption in endogenous pain modulation [58]. Therefore, more studies involving potential confounders and sufficient sample sizes are required in order to further elucidate the factors associated with CPM response.

## Figures and Tables

**Table 1:** Sex-wise differences in characteristics of the study participants.

	Men	Women	*p* value
	*N* = 43	*N* = 43	
Age (years)	20.8 (0.8)	21.0 (0.8)	0.289
Height (cm)	172.2 (5.4)	158.1 (6.1)	<0.001^*∗*^
Weight (kg)	66.3 (9.8)	52.0 (5.8)	<0.001^*∗*^
BMI (kg/m^2^)	22.4 (3.3)	20.8 (2.1)	0.010^*∗*^
BMR (kcal)	1657.6 (159.9)^#^	1273.3 (96.3)^#^	<0.001^*∗*^
MVPA (METs × h/week)	14.8 (6.3)	13.7 (5.0)	0.342
Baseline PPT (N)	20.0 (13.6)^#^	16.5 (12.5)^#^	0.019^*∗*^
CPM response	0.24 (0.28)^#^	0.24 (0.37)^#^	0.948
CPM response immediately after CPM	0.11 (0.10)	0.15 (0.33)	0.144

Values: mean (standard deviation) or median (interquartile  range)^#^; CPM response = (PPT during conditioning stimulus − baseline PPT)/baseline PPT; CPM response immediately after CPM = (PPT immediately after conditioning stimulus − baseline PPT)/baseline PPT; comparison between the two groups was performed using Student's *t*-test or Mann–Whitney *U* test, depending on the distribution of data. ^*∗*^Statistical significance (*p* < 0.05); BMI: body mass index, BMR: basal metabolic rate, MVPA: moderate-to-vigorous physical activity, PPT: pressure pain threshold, CPM: conditioned pain modulation.

**Table 2:** Results of multiple regression analysis with PPT as a dependent variable.

	Variable	Adjusted *R*^2^	*B*	*β*	*p* value	95% CI for *B*	
						Lower limit	Upper limit
Overall		0.054					
	BMR		0.012	0.256	0.018	0.002	0.022
Men		—					
	None						
Women		—					
	None						

*B*: unstandardized coefficient, *β*: standardized coefficient, PPT: pressure pain threshold, CI: confidence interval, BMR: basal metabolic rate.

**Table 3:** Results of multiple regression analysis with CPM response as a dependent variable.

	Variable	Adjusted *R*^2^	*B*	*β*	*p* value	95% CI for *B*	
						Lower limit	Upper limit
Overall		—					
	None						
Men		—					
	None						
Women		0.137					
	MVPA		0.020	0.397	0.008	0.005	0.035

*B*: unstandardized coefficient, *β*: standardized coefficient, CPM: conditioned pain modulation, CI, confidence interval, MVPA, moderate-to-vigorous physical activity.

## References

[1] Yarnitsky D., Arendt-Nielsen L., Bouhassira D. (2010). Recommendations on terminology and practice of psychophysical DNIC testing. *European Journal of Pain*.

[2] Yarnitsky D. (2010). Conditioned pain modulation (the diffuse noxious inhibitory control-like effect): its relevance for acute and chronic pain states. *Current Opinion in Anaesthesiology*.

[3] Popescu A., Leresche L., Truelove E. L., Drangsholt M. T. (2010). Gender differences in pain modulation by diffuse noxious inhibitory controls: A systematic review. *Pain*.

[4] Bulls H. W., Freeman E. L., Anderson A. J., Robbins M. T., Ness T. J., Goodin B. R. (2015). Sex differences in experimental measures of pain sensitivity and endogenous pain inhibition. *Journal of Pain Research*.

[5] Naugle K. M., Riley 3rd J. L. (2014). Self-reported physical activity predicts pain inhibitory and facilitatory function. *Medicine and Science in Sports and Exercise*.

[6] Daenen L., Nijs J., Roussel N., Wouters K., Van Loo M., Cras P. (2013). Dysfunctional pain inhibition in patients with chronic whiplash-associated disorders: An experimental study. *Clinical Rheumatology*.

[7] Vaegter H. B., Handberg G., Graven-Nielsen T. (2016). Hypoalgesia after Exercise and the Cold Pressor Test is Reduced in Chronic Musculoskeletal Pain Patients with High Pain Sensitivity. *Clinical Journal of Pain*.

[8] Fillingim R. B. (2000). Sex, gender, and pain: women and men really are different. *Current Review of Pain*.

[9] Mogil J. S. (2012). Sex differences in pain and pain inhibition: multiple explanations of a controversial phenomenon. *Nature Reviews Neuroscience*.

[10] Racine M., Tousignant-Laflamme Y., Kloda L. A., Dion D., Dupuis G., Choinire M. (2012). A systematic literature review of 10 years of research on sex/sex and experimental pain perception – part 1: are there really differences between women and men?. *Pain*.

[11] Racine M., Tousignant-Laflamme Y., Kloda L. A., Dion D., Dupuis G., Choinire M. (2012). A systematic literature review of 10 years of research on sex/gender and pain perception—part 2: do biopsychosocial factors alter pain sensitivity differently in women and men?. *Pain*.

[12] Granot M., Weissman-Fogel I., Crispel Y. (2008). Determinants of endogenous analgesia magnitude in a diffuse noxious inhibitory control (DNIC) paradigm: Do conditioning stimulus painfulness, gender and personality variables matter?. *Pain*.

[13] France C. R., Suchowiecki S. (1999). A comparison of diffuse noxious inhibitory controls in men and women. *Pain*.

[14] Baad-Hansen L., Poulsen H. F., Jensen H. M., Svensson P. (2005). Lack of sex differences in modulation of experimental intraoral pain by diffuse noxious inhibitory controls (DNIC). *Pain*.

[15] Landmark T., Romundstad P., Borchgrevink P. C., Kaasa S., Dale O. (2011). Associations between recreational exercise and chronic pain in the general population: Evidence from the HUNT 3 study. *Pain*.

[16] Matsubara T., Arai Y. C., Shimo K. (2010). Effects of cognitive-behavioral therapy on pain intensity and level of physical activity in Japanese patients with chronic pain- a preliminary quasi-experimental study. *Journal of Physical Therapy Science*.

[17] Pinto R. Z., Ferreira P. H., Kongsted A., Ferreira M. L., Maher C. G., Kent P. (2014). Self-reported moderate-to- vigorous leisure time physical activity predicts less pain and disability over 12 months in chronic and persistent low back pain. *European Journal of Pain*.

[18] Koes B. W., Van Tulder M., Lin C.-W. C., Macedo L. G., McAuley J., Maher C. (2010). An updated overview of clinical guidelines for the management of non-specific low back pain in primary care. *European Spine Journal*.

[19] Naugle K. M., Fillingim R. B., Riley III J. L. (2012). A meta-analytic review of the hypoalgesic effects of exercise. *Journal of Pain*.

[20] Verbunt J. A., Seelen H. A., Vlaeyen J. W. (2003). Disuse and deconditioning in chronic low back pain: concepts and hypotheses on contributing mechanisms. *European Journal of Pain*.

[21] Andrzejewski W., Kassolik K., Brzozowski M., Cymer K. (2010). The influence of age and physical activity on the pressure sensitivity of soft tissues of the musculoskeletal system. *Journal of Bodywork and Movement Therapies*.

[22] Ellingson L. D., Colbert L. H., Cook D. B. (2012). Physical activity is related to pain sensitivity in healthy women. *Medicine and Science in Sports and Exercise*.

[23] Caspersen C. J., Pereira M. A., Curran K. M. (2000). Changes in physical activity patterns in the United States, by sex and cross-sectional age. *Medicine & Science in Sports & Exercise*.

[24] Jensen K. B., Kosek E., Petzke F. (2009). Evidence of dysfunctional pain inhibition in Fibromyalgia reflected in rACC during provoked pain. *Pain*.

[25] McLoughlin M. J., Colbert L. H., Stegner A. J., Cook D. B. (2011). Are women with fibromyalgia less physically active than healthy women?. *Medicine and Science in Sports and Exercise*.

[26] Ellingson L. D., Shields M. R., Stegner A. J., Cook D. B. (2012). Physical activity, sustained sedentary behavior, and pain modulation in women with fibromyalgia. *Journal of Pain*.

[27] McLoughlin M. J., Stegner A. J., Cook D. B. (2011). The relationship between physical activity and brain responses to pain in fibromyalgia. *Journal of Pain*.

[28] Azevedo M. R., Araújo C. L. P., Reichert F. F., Siqueira F. V., da Silva M. C., Hallal P. C. (2007). Gender differences in leisure-time physical activity. *International Journal of Public Health*.

[29] Melzer K., Heydenreich J., Schutz Y., Renaud A., Kayser B., Mäder U. (2016). Metabolic equivalent in adolescents, active adults and pregnant women. *Nutrients*.

[30] Roberts D., Gebhardt D. L., Gaskill S. E., Roy T. C., Sharp M. A. (2016). Current considerations related to physiological differences between the sexes and physical employment standards. *Applied Physiology, Nutrition, and Metabolism*.

[31] Lowe J. C., Yellin J., Honeyman-Lowe G. (2006). Female fibromyalgia patients: lower resting metabolic rates than matched healthy controls. *Medical Science Monitor*.

[32] Okifuji A., Hare B. D. (2015). The association between chronic pain and obesity. *Journal of Pain Research*.

[33] Paley C. A., Johnson M. I. (2016). Physical activity to reduce systemic inflammation associated with chronic pain and obesity: a narrative review. *Clinical Journal of Pain*.

[34] Price R. C., Asenjo J. F., Christou N. V., Backman S. B., Schweinhardt P. (2013). The role of excess subcutaneous fat in pain and sensory sensitivity in obesity. *European Journal of Pain (United Kingdom)*.

[35] Morris M. C., Walker L., Bruehl S., Hellman N., Sherman A. L., Rao U. (2015). Race Effects on Conditioned Pain Modulation in Youth. *Journal of Pain*.

[36] Riley 3rd J. L., Cruz-Almeida Y., Glover T. L. (2014). Age and race effects on pain sensitivity and modulation among middle-aged and older adults. *Journal of Pain*.

[37] Washington L. L., Gibson S. J., Helme R. D. (2000). Age-related differences in the endogenous analgesic response to repeated cold water immersion in human volunteers. *Pain*.

[38] Edwards R. R., Fillingim R. B., Ness T. J. (2003). Age-related differences in endogenous pain modulation: A comparison of diffuse noxious inhibitory controls in healthy older and younger adults. *Pain*.

[39] Riley 3rd J. L., Robinson M. E., Wise E. A., Myers C. D., Fillingim R. B. (1998). Sex differences in the perception of noxious experimental stimuli: A meta-analysis. *Pain*.

[40] Kennedy D. L., Kemp H. I., Ridout D., Yarnitsky D., Rice A. S. (2016). Reliability of conditioned pain modulation: a systematic review. *PAIN*.

[41] Faul F., Erdfelder E., Buchner A., Lang A.-G. (2009). Statistical power analyses using G∗Power 3.1: tests for correlation and regression analyses. *Behavior Research Methods*.

[42] Harris J. A., Benedict F. G. (1918). A biometric study of human basal metabolism. *Proceedings of the National Academy of Sciences of the United States of America*.

[43] Schneider P. L., Crouter S. E., Lukajic O., Bassett D. R. (2003). Accuracy and reliability of 10 pedometers for measuring steps over a 400-m walk. *Medicine and Science in Sports and Exercise*.

[44] Kumahara H., Schutz Y., Ayabe M. (2004). The use of uniaxial accelerometry for the assessment of physical-activity-related energy expenditure: A validation study against whole-body indirect calorimetry. *British Journal of Nutrition*.

[45] Pud D., Granovsky Y., Yarnitsky D. (2009). The methodology of experimentally induced diffuse noxious inhibitory control (DNIC)-like effect in humans. *Pain*.

[46] van Wijk G., Veldhuijzen D. S. (2010). Perspective on diffuse noxious inhibitory controls as a model of endogenous pain modulation in clinical pain syndromes. *Journal of Pain*.

[47] Vaegter H. B., Handberg G., Graven-Nielsen T. (2014). Similarities between exercise-induced hypoalgesia and conditioned pain modulation in humans. *Pain*.

[48] Vaegter H. B., Handberg G., Jørgensen M. N., Kinly A., Graven-Nielsen T. (2015). Aerobic Exercise and Cold Pressor Test Induce Hypoalgesia in Active and Inactive Men and Women. *Pain Medicine (United States)*.

[49] Cymet T. C. (2003). A practical approach to fibromyalgia. *Journal of the National Medical Association*.

[50] Yi J., Zheng J.-Y., Zhang W., Wang S., Yang Z.-F., Dou K.-F. (2014). Decreased pain threshold and enhanced synaptic transmission in the anterior cingulate cortex of experimental hypothyroidism mice. *Molecular Pain*.

[51] Hermans L., Van Oosterwijck J., Goubert D. (2016). Inventory of Personal Factors Influencing Conditioned Pain Modulation in Healthy People: A Systematic Literature Review. *Pain Practice*.

[52] Skovbjerg S., Jørgensen T., Arendt-Nielsen L., Ebstrup J. F., Carstensen T., Graven-Nielsen T. (2017). Conditioned Pain Modulation and Pressure Pain Sensitivity in the Adult Danish General Population: The DanFunD Study. *Journal of Pain*.

[53] Umeda M., Lee W., Marino C. A., Hilliard S. C. (2016). Influence of moderate intensity physical activity levels and gender on conditioned pain modulation. *Journal of sports sciences*.

[54] Naugle K. M., Ohlman T., Naugle K. E., Riley Z. A., Keith N. R. (2017). Physical activity behavior predicts endogenous pain modulation in older adults. *Pain*.

[55] Weissman-Fogel I., Sprecher E., Pud D. (2008). Effects of catastrophizing on pain perception and pain modulation. *Experimental Brain Research*.

[56] Altman N., Krzywinski M. (2015). Points of Significance: Association, correlation and causation. *Nature Methods*.

[57] Hikihara Y., Tanaka S., Ohkawara K., Ishikawa-Takata K., Tabata I. (2012). Validation and comparison of 3 accelerometers for measuring physical activity intensity during nonlocomotive activities and locomotive movements. *Journal of Physical Activity and Health*.

[58] Goodin B. R., McGuire L., Allshouse M. (2009). Associations Between Catastrophizing and Endogenous Pain-Inhibitory Processes: Sex Differences. *Journal of Pain*.

